# Small infrarenal aortic diameter associated with lower-extremity peripheral artery disease in Chinese hypertensive adults

**DOI:** 10.1038/s41598-017-12587-x

**Published:** 2017-11-06

**Authors:** Jie Liu, Xin Jia, Senhao Jia, Xianhui Qin, Tao Zhang, Lishun Liu, Haibo Li, Dan Rong, Ziyi Zhou, Yuxiang Song, Shangwei Zuo, Chen Duan, Zhongyin Wu, Ren Wei, Yangyang Ge, Xian Wang, Wei Kong, Xiping Xu, Raouf A. Khalil, Yong Huo, Wei Guo

**Affiliations:** 1 Department of Vascular and Endovascular Surgery, Chinese PLA General Hospital, Beijing, China; 2 Vascular Surgery Research Laboratories, Division of Vascular and Endovascular Surgery, Brigham and Women’s Hospital, Harvard Medical School, Boston, Massachusetts, United States; 3 National Center for Clinical Research in Kidney Disease, Guangdong Institute of Nephrology, Southern Medical University, Guangzhou, China; 4 Department of Vascular Surgery, Peking University People’s Hospital, Beijing, China; 5 Institute of Biomedicine, Anhui Medical University, Hefei, China; 6 Department of Physiology and Pathophysiology, School of Basic Medical Sciences, Peking University, Beijing, China; 7 Key Laboratory of Molecular Cardiovascular Science, Ministry of Education, Beijing, China; 8 Department of Cardiology, Peking University First Hospital, Beijing, China

## Abstract

Several studies suggest that infrarenal aortic diameter is associated with lower-extremity peripheral artery disease (LE-PAD). However, data regarding the associations between infrarenal aortic diameter and LE-PAD are limited, especially in large sample populations and Asian or Chinese populations. Our analysis included 17279 Chinese hypertensive adults comprising 6590 men and 10689 women with a mean age of 64.74 ± 7.41 years. Participants were selected from 22693 candidates from two large population-based cohort-studies. The primary noninvasive test for diagnosis of LE-PAD is the ankle–brachial index (ABI) at rest and typically an ABI ≤ 0.90 is used to define LE-PAD. The prevalence of LE-PAD was found to significantly decrease as the aortic diameter increased according to the tertile of the aortic diameter. LE-PAD was significantly more prevalent in the lowest tertile (OR = 1.58, 95% CI = 1.29–1.94, p < 0.001) and similarly prevalent in the highest tertile (OR = 0.92, 95% CI = 0.73–1.16, p = 0.49) when compared with the median tertile. No significant interactions between the aortic diameter and any of the stratified variables were found (all p > 0.05). In conclusion, Small aortic diameter (as opposed to large aortic diameter) is significantly associated with LE-PAD in Chinese hypertensive adults.

## Introduction

Traditionally, infrarenal aortic diameter has only been of clinical interest when examined in the context of abdominal aortic aneurysms (AAAs)^1^. However, studies^2–4^ have shown that the infrarenal aorta plays an important role in predicting the risk of cardiovascular events and mortality.

Several studies have further suggested that the infrarenal aortic diameter is associated with lower-extremity peripheral artery disease (LE-PAD)^2,4–6^ in patients with AAA as well as those with small or enlarged non-aneurysmal aortas. However, the available data regarding the associations between infrarenal aortic diameter and LE-PAD are limited, especially in large and Asian or Chinese populations.

The aim of this study was to assess the relationship between the infrarenal aortic diameter and LE-PAD in a large population of over 17000 Chinese hypertensive adult subjects.

## Methods

### Study participants

Prospective participants for this study were selected from candidates who had taken part in two large population-based studies that were conducted between May 19th, 2008, and August 24th, 2013. These studies investigated the clinical presentation and management of hypertension in China. Both studies used the same inclusion and exclusion criteria for participant selection as well as the same follow-up schedules. The first study was the China Stroke Primary Prevention Trial (CSPPT), (clinicaltrials.gov identifier: NCT00794885). Patients were eligible when they had hypertension and were adult men or women aged 45 to 75 years. Hypertension was defined as systolic blood pressure ≥140 mmHg or diastolic blood pressure ≥90 mmHg in seated resting position or were taking antihypertensive medication. The major exclusion criteria included history of stroke diagnosed by a physician, myocardial infarction (MI), heart failure, coronary revascularization, or congenital heart disease^7^. Further details regarding inclusion and exclusion criteria, treatment assignment, and outcome measures of this trial have been previously described (http://clinicaltrials.gov/ct2/show/NCT00794885). The second cohort of patients were enrolled from the same regions and followed the same protocol as the first, with the only exception being that subjects did not strictly need to be treated with the designated drugs used in the trial. At the final follow-up visit in these two large cohort studies, which took place in July and August 2013 in 32 communities in the Jiangsu and Anhui provinces of China, aortic diameter measurements were obtained with the aim of investigating abdominal aortic diameter and the prevalence of AAAs in a large hypertensive population in China.

**Figure 1:**
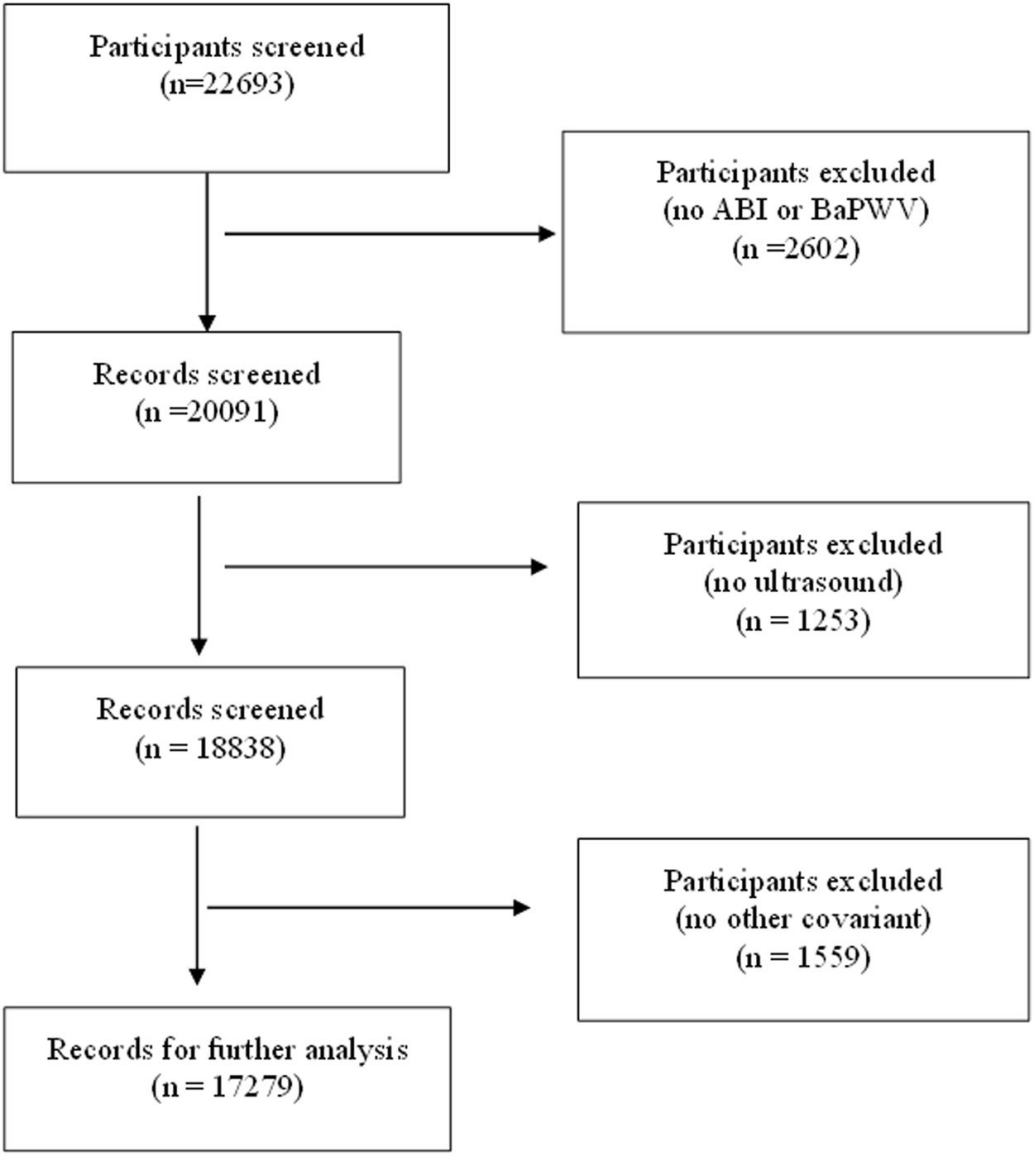
Flow diagram of the screening and enrollment of study participants. ABI, ankle–brachial index; BaPWV, brachial-ankle pulse wave velocity.

The medical charts of 22693 candidates were screened and 2602 were excluded as ABI and BaPWV measurements were not available. Furthermore, 1253 candidates were excluded as measurement of the abdominal aorta by ultrasound was not provided, while information on covariant factors was not available for a further 1559 candidates. These included sex; age; body mass index (BMI); MTHFR C677T polymorphisms; heart rate; systolic blood pressure (SBP); diastolic blood pressure (DBP); cigarette smoking; folic acid supplementation; and laboratory results for total cholesterol (TC), triglycerides (TG), high-density lipoprotein cholesterol (HDL-C), fasting glucose, creatinine, uric acid, and homocysteine. After screening, the data for 17279 participants was entered for further analysis. The flow chart of this screening process is presented in Fig. 1.

This study was conducted in accordance with the principles of the Declaration of Helsinki and approved by the Human Subject Committee at the Biomedical Institute of Anhui Medical University, Hefei, China (FWA assurance number FWA00001263). Written and informed consent was obtained from each participant prior to data collection.

### Evaluation of the abdominal aorta

The diameter of the infrarenal aorta was measured using ultrasound and the measurement was performed by experienced technicians using a professional ultrasound machine with a 3.5-MHz real-time sector scanner (SonoScape Technologies, Shenzhen, China). After a longitudinal scan of the aorta (extending from the level of the renal arteries to the aortic bifurcation), the diameter of the abdominal aorta was measured between two reference points. The first point was localized just 10 mm inferior to the level of the renal arteries and recorded as the infrarenal aortic diameter, while the second point was just 10 mm superior to the aortic bifurcation and was recorded as the distal aortic diameter. If an obvious dilation of the aorta was identified between these two points, the diameter was measured at its largest point and recorded as the maximal diameter of the infrarenal aorta. AAA was the maximal diameter of the infrarenal aorta ≥30 mm^8^.

In addition, inter and intra-observer measurement reliability was assessed. In order to determine reliability, 100 cases from the entire study population were randomly sampled with the aim of selecting approximately equal numbers of men and women. Inter -and intra-observer agreement was assessed using the methods proposed by Bland and Altman^9^. No significant differences were observed either within (intra) or between (inter) observers^10^.

### Ankle-brachial index and brachial-ankle pulse wave velocity measurements

Participants were at rest ≥10 min before measurements were taken. The ankle-brachial index (ABI) and the brachial-ankle pulse wave velocity (BaPWV) were automatically measured with form PWV/ABI instruments (form PWV/ABI, BP-203RPE; Omron-Colin, Japan) as previously described^11–13^. These measurements were taken by trained volunteers who were recruited from local medical colleges. The ABI for each leg was calculated by dividing the SBP obtained at the ankle level by the SBP of the brachial artery. The data regarding validity and reproducibility of this automatic device has been previously published^14,15^. An ABI ≤ 0.90 in either leg was used to define LE-PAD^16^.

### Data collection and laboratory tests

A standard questionnaire was administered by trained staff in order to obtain information on demographic characteristics, personal and family medical history, and lifestyle risk factors (such as cigarette and alcohol consumption). The interview included questions related to the diagnosis and treatment of hypertension, diabetes, dyslipidemia, stroke, and cardiovascular events.

A venous blood sample was obtained from each participant after 12 to 15 hours of fasting. Serum or plasma samples were separated within 30 min and stored at −70 °C. Concentrations of fasting glucose, creatinine, uric acid, homocysteine and lipids (including total TC, HDL-C, and TG) were measured using automatic clinical analyzers (Beckman Coulter) at the core laboratory of the National Clinical Research Center for Kidney Disease at Nanfang Hospital, Guangzhou, China.

### Statistical analysis

Data are presented as the mean ± standard deviation (SD) or as the median (range) for continuous variables and proportions (percentages) for categorical variables. Inter-group comparisons were performed using a two-sample t-test for continuous variables and a chi-squared test for categorical variables.

Logistic regression analysis was used to estimate the odds ratios (ORs) and 95% confidence intervals (CIs) for risk of LE-PAD with increasing tertiles of distal aortic diameter, using the median tertile as the reference group. Results were adjusted for age, sex and BMI in model I. To investigate independent association, we further adjusted for heart rate, systolic blood pressure, diastolic blood pressure, cigarette smoking, folic acid supplementation, level of total cholesterol, triglycerides, HDL-C, fasting glucose, creatinine, uric acid, homocysteine, and MTHFR C677T polymorphisms in model II. Analyses were stratified by sex, age, BMI, MTHFR C677T polymorphisms, cigarette smoking, folic acid supplementation in order to examine the effect of these factors on the above associations. Tests for effect modification by pre-specified subgroups used interaction terms between subgroup indicators and tertiles of the aortic diameter by the likelyhood ratio test. We further ran a sensitive analysis to exclude participants with an AAA or ABI ≥ 1.40 and replaced the distal aortic diameter with either the maximal diameter of the infrarenal aorta or the infrarenal aortic diameter, in order to assess whether or not the above associations would be modified. Tests for trends were computed by modeling the medians of the aortic diameter tertiles as continuous variables. A P-value < 0.05 was considered statistically significant.

All analyses were performed using the statistical software packages R (http://www.R-project.org, The R Foundation) and EmpowerStats (www.empowerstats.com, X&Y solutions, Inc., Boston, MA). A two-sided significance level of 0.05 was used to evaluate statistical significance.

## Results

### Study population characteristics

The descriptive characteristics of the 17279 participants included in our analysis are presented in Table 1 and are reported as the total study sample as well as by the presence of LE-PAD (607 participants [3.51%]). The study group was comprised of 6590 men and 10689 women with a mean age of 64.74 ± 7.41 years. When compared to non-LE-PAD participants, the participants in the LE-PAD group were significant older and the following variables were found to be significantly higher in the LE-PAD group: body mass index (BMI), heart rate; proportion of current smokers; levels of TC, triglycerides, fasting glucose, creatinine, uric acid, and homocysteine; and brachial-ankle pulse wave velocity (all p < 0.01). However, the LE-PAD group did have significantly lower diastolic blood pressure (DPB), levels of high-density lipoprotein cholesterol (HDL-C), as well as a smaller distal aortic diameter (all p < 0.001)

**Table 1:** Characteristics of the study population.

Characteristics	Overall n = 17279	LE-PAD group n = 607	Control group n = 16683	P-value
Male	6590 (38.10%)	222 (36.60%)	6368 (38.20%)	0.42
Age, yr	64.74 ± 7.41	68.29 ± 7.77	64.61 ± 7.36	<0.001
BMI, kg/m^2^	24.84 ± 3.85	25.72 ± 4.73	24.80 ± 3.81	<0.001
MTHFR C677T polymorphisms				0.36
CC	4834 (28.00%)	163 (26.90%)	4671 (28.00%)	
CT	8427 (48.80%)	313 (51.60%)	8114 (48.70%)	
TT	4018 (23.30%)	131 (21.60%)	3887 (23.30%)	
Heart rate, beats/min	76.88 ± 11.49	80.92 ± 13.96	76.73 ± 11.36	<0.001
Systolic blood pressure, mmHg	136.87 ± 17.98	138.13 ± 22.20	136.83 ± 17.80	0.08
Diastolic blood pressure, mmHg	82.07 ± 11.03	79.47 ± 12.01	82.17 ± 10.98	<0.001
Cigarette smoking				0.002
Never	11861 (68.60%)	388 (63.90%)	11473 (68.80%)	
Former	1877 (10.90%)	60 (9.90%)	1817 (10.90%)	
Current	3541 (20.50%)	159 (26.20%)	3382 (20.30%)	
Folic acid supplementation				0.76
No	10488 (60.70%)	372 (61.30%)	10116 (60.70%)	
Yes	6791(39.30%)	235 (38.70%)	6556 (39.30%)	
Laboratory results				
Total cholesterol, mmol/L	5.28 ± 1.09	5.58 ± 1.14	5.27 ± 1.08	<0.001
Triglycerides, mmol/L	1.78 ± 1.39	2.10 ± 1.46	1.76 ± 1.39	<0.001
HDL-C, mmol/L	1.27 ± 0.31	1.21 ± 0.27	1.28 ± 0.31	<0.001
Fasting glucose, mmol/L	6.25 ± 1.99	6.96 ± 3.11	6.23 ± 1.93	<0.001
Creatinine, μmol/L	68.41 ± 26.46	72.86 ± 27.08	68.25 ± 26.42	<0.001
Uric acid, mmol/L	328.62 ± 89.89	352.02 ± 96.87	327.77 ± 89.52	<0.001
Homocysteine, μmol/L	13.61 ± 7.18	15.37 ± 7.95	13.55 ± 7.14	<0.001
ABI	1.07 ± 0.10	0.77 ± 0.18	1.08 ± 0.07	<0.001
BaPWV, cm/s	1732.80 ± 408.47	1805.34 ± 593.39	1730.16 ± 399.91	<0.001
Distal aortic diameter, mm	12.98 ± 2.04	12.31 ± 2.14	13.00 ± 2.03	<0.001

BMI, Body mass index; LE-PAD, lower-extremity peripheral artery disease; CSPPT, China Stroke Primary Prevention Trial; HDL-C, high-density lipoprotein cholesterol; ABI, ankle–brachial index; BaPWV, brachial-ankle pulse wave velocity.

### Proportion of LE-PAD according to the tertile of the distal aortic diameter

The proportion of LE-PAD according to the tertile of the distal aortic diameter in both the overall and subgroups is presented in Table 2. The median (range) distal aortic diameter for men was 11.90 (4.70–12.90) mm in the lowest tertile, 13.80 (13.00–14.55) mm in the median tertile, and 15.60 (14.60–51.30) mm in the highest tertile. The median (range) distal aortic diameter for women was 10.60 (5.20–11.50) mm in the lowest tertile, 12.30 (11.60–13.15) mm in the median tertile, and 14.10 (13.20–30.50) mm in the highest tertile. In the overall population, the proportion of LE-PAD significantly decreased as the distal aortic diameter increased according to the tertile of the distal aortic diameter (lowest tertile vs. median tertile vs. highest tertile: 5.20% vs. 2.80% vs. 2.60%, respectively. p < 0.001, p value for trend <0.0001). In the subgroups for sex, age, BMI, MTHFR C677T genotype, cigarette smoking and folic acid supplementation, the trend for the proportion of LE-PAD was a similar and significant decrease according to the tertile of the distal aortic diameter.

**Table 2:** Proportion of LE-PAD according to tertile of distal aortic diameter in overall group and subgroups.

	Lowest tertile	Median tertile	Highest tertile	P-value	P-value for trend
Overall	288 (5.20%)	164 (2.80%)	155 (2.60%)	<0.001	<0.0001
Sex					
male	112 (5.10%)	54 (2.60%)	56 (2.40%)	<0.001	<0.0001
female	176 (5.30%)	110 (3.00%)	99 (2.70%)	<0.001	<0.0001
Age, yr					
<65	78 (2.90%)	62 (2.10%)	63 (2.00%)	0.03	<0.0001
≥65	210 (7.40%)	102 (3.70%)	92 (3.30%)	<0.001	<0.0001
Body mass index, kg/m2					
<25	161 (5.10%)	70 (2.20%)	59 (2.00%)	<0.001	<0.0001
≥25	127 (5.40%)	94 (3.60%)	96 (3.10%)	<0.001	<0.0001
MTHFR C677T polymorphisms					
CC	83 (5.30%)	38 (2.40%)	42 (2.50%)	<0.001	<0.0001
CT	149 (5.50%)	86 (3.00%)	78 (2.70%)	<0.001	<0.0001
TT	56 (4.40%)	40 (3.00%)	35 (2.50%)	<0.001	<0.0001
Cigarette smoking					
Never	174 (4.80%)	112 (2.70%)	102 (2.50%)	<0.001	<0.0001
Former	35 (5.80%)	13 (2.30%)	12 (1.70%)	<0.001	<0.0001
Current	79 (6.20%)	39 (3.50%)	41 (3.50%)	0.003	<0.0001
Folic acid supplementation					
No	179 (5.30%)	106 (3.00%)	87 (2.40%)	<0.001	<0.0001
Yes	109 (5.10%)	58 (2.60%)	68 (2.80%)	<0.001	<0.0001

LE-PAD, lower-extremity peripheral artery disease. CSPPT, China Stroke Primary Prevention Trial; Distal Aortic Diameter, median (range), mm. Men: lowest tertile, 11.90 (4.70–12.90) mm; median tertile, 13.80 (13.00–14.55) mm; 15.60 (14.60–51.30); highest tertile, 15.60 (14.60–51.30) mm. Women: lowest tertile, 10.60 (5.20–11.50) mm; median tertile, 12.30 (11.60–13.15) mm; highest tertile, 14.10 (13.20–30.50) mm.

### Relationship between the distal aortic diameter and LE-PAD

**Figure 2:**
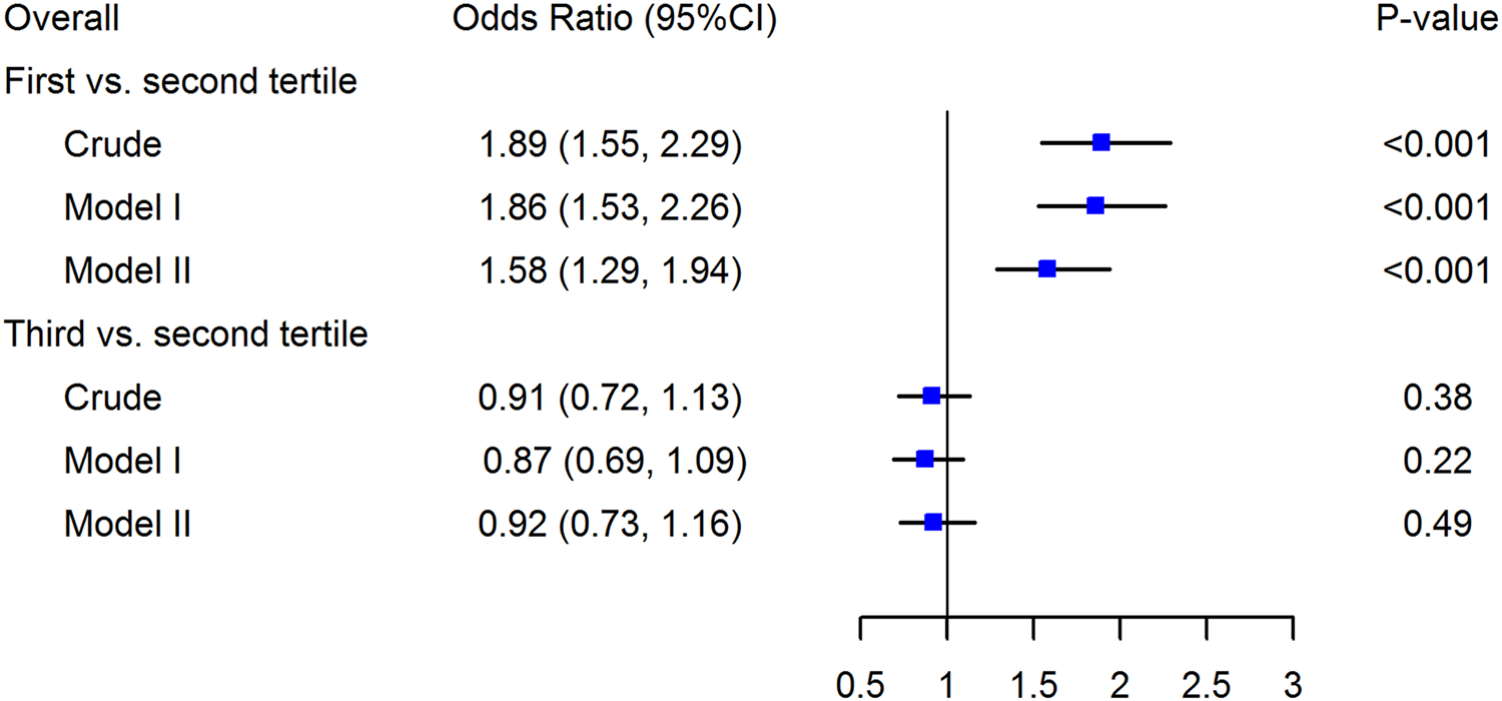
Forrest plot for LE-PAD in relation to tertiles of the aortic diameter in the whole population. CI, confidence interval. LE-PAD, lower-extremity peripheral artery disease. Crude, not adjusted; Model I adjusted for sex, age and body mass index; Model II adjusted for sex, age, body mass index, heart rate, systolic blood pressure, diastolic blood pressure, cigarette smoking, folic acid supplementation, level of total cholesterol, triglycerides, HDL-C, fasting glucose, creatinine, uric acid, homocysteine, and MTHFR C677T polymorphisms.

The ORs reflecting the strength of the association between the aortic diameter and LE-PAD in various multivariate logistic regression models for overall participants are presented in Fig. 2 and Table S1 in Supplement 1. LE-PAD was more prevalent in the lowest tertile when compared with the median tertile, the crude OR was 1.89 (95% CI, 1.55–2.29, p < 0.001) and ORs were similar in models I and II (model I, OR = 1.86, 95% CI, 1.53–2.26, p < 0.001; model II, OR = 1.58, 95% CI, 1.29–1.94, p < 0.001). When compared with that of the median tertile, LE-PAD was similarly prevalent in the highest tertile, the crude OR was 0.91 (95% CI, 0.72–1.13, p = 0.38), and ORs were similar in models I and II (model I, OR = 0.87, 95% CI, 0.69–1.09, p = 0.22; model II, OR = 0.92, 95% CI, 0.73–1.16, p = 0.49)

**Figure 3:**
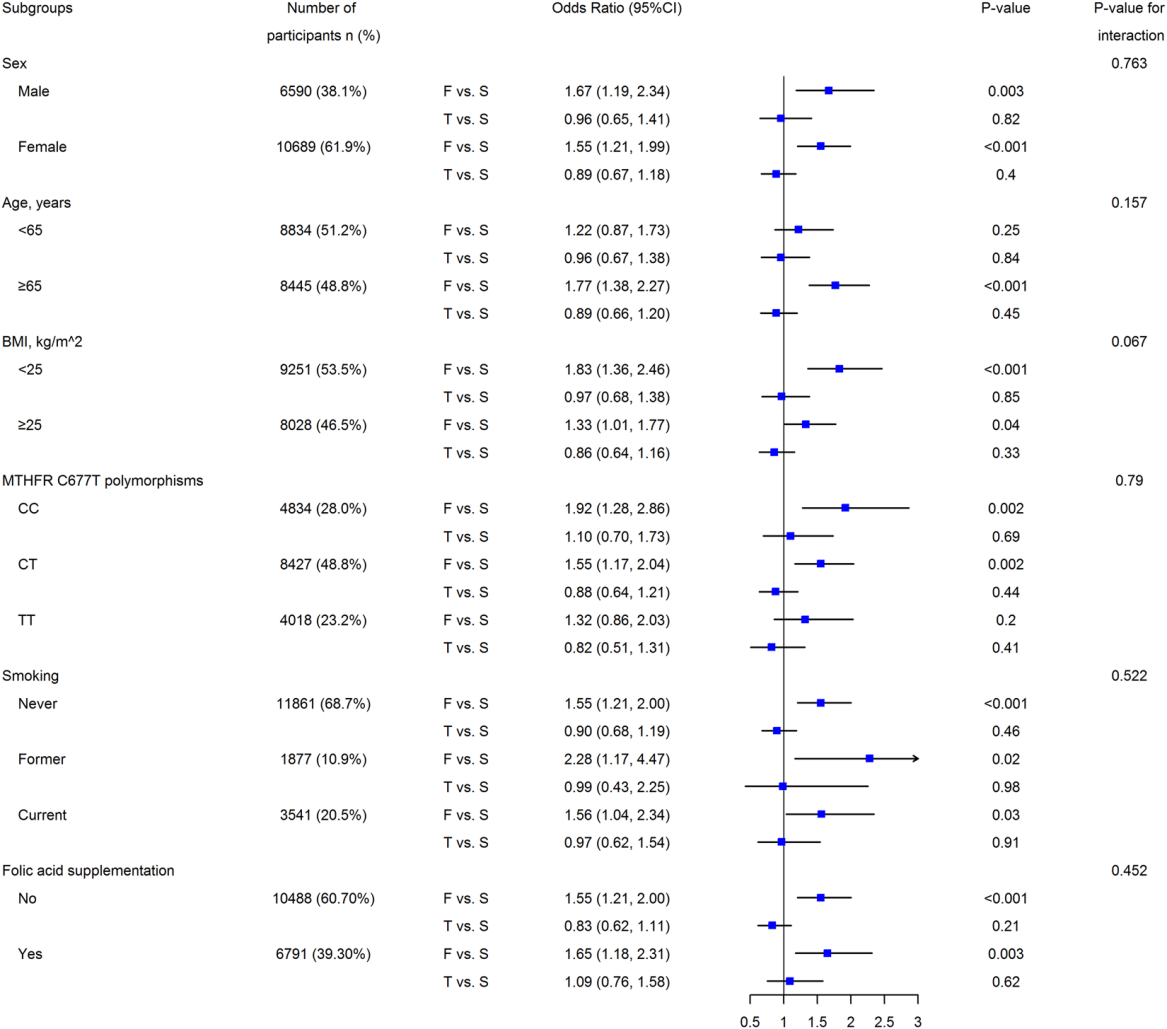
Forrest plot for LE-PAD in relation to tertiles of the aortic diameter according to baseline characteristics. LE-PAD, lower-extremity peripheral artery disease; BMI, Body mass index. F vs. S, First vs. second tertile of aortic diameter. T vs. S, Third vs. second tertile of aortic diameter. CI, confidence interval. The regression analysis was adjusted for sex, age, body mass index, heart rate, systolic blood pressure, diastolic blood pressure, cigarette smoking, folic acid supplementation, level of total cholesterol, triglycerides, HDL-C, fasting glucose, creatinine, uric acid, homocysteine, and MTHFR C677T polymorphisms.

Subgroup analysis of the association between the aortic diameter and LE-PAD in a multivariate logistic regression model is presented in Fig. 3 and Table S2 in Supplement 1. Compared with that of the median tertile, the lowest tertile increased the risk for LE-PAD in all subgroups. Compared with that of the median tertile, the highest tertile did not significantly increase or decrease the risk for LE-PAD in all subgroups.

No significant interactions were found between aortic diameter and any of the stratified variables in relation to LE-PAD; including sex, age, BMI, MTHFR C677T polymorphisms, cigarette smoking, and folic acid supplementation (Table S2 in Supplement 1, p > 0.05).

The results of the sensitive analysis to exclude the participants with an AAA or ABI ≥ 1.40 showed that the association between the aortic diameter and LE-PAD was not changed. Furthermore, when the distal aortic diameter was replaced with either the maximal diameter of the infrarenal aorta or the infrarenal aortic diameter, the association between the aortic diameter and LE-PAD was not altered (data not shown).

## Discussion

In this observational study of 17279 Chinese hypertensive adults, the prevalence of LE-PAD was found to be 3.51% and the proportion of LE-PAD significantly decreased as the aortic diameter increased according to the tertile of the aortic diameter. LE-PAD was significantly more prevalent in the lowest tertile and similarly prevalent in the highest tertile when compared with the median tertile. No significant interactions were found between the aortic diameter and any of the stratified variables. Our findings suggest that the small aortic diameter (as opposed to the large aortic diameter) is associated with LE-PAD in Chinese hypertensive adults.

Several studies have previously demonstrated an association between the aortic diameter and PAD^2,4–6^. A study^5^ of 54 elderly people was the first to observe a negative relationship between the abdominal aortic diameter and the calf: brachial systolic pressure ratio, suggesting that a smaller aortic diameter was associated with a higher prevalence of PAD. In a cross-sectional study^6^ of 1572 patients aged between 18 and 79 years with risk factors of or clinically manifested vascular disease, Bosch *et al*. found that both small and large aortic diameters were associated with PAD. When compared with patients with a normal aortic diameter (median tertile), PAD was twice as prevalent in patients at both the lowest and highest tertiles. The Health in Men Study^4^ further showed a J-shaped relationship between aortic diameter and prevalent claudication. For prevalent claudication, with 19 to 22.9mm used as the reference range, the adjusted OR was 1.24 (95% CI, 0.95–1.62), 1.44 (95% CI, 1.18–1.75), and 1.53 (95% CI, 1.17–2.00) with aortic diameters of <19 mm, 23 to 29.9 mm, and ≥30 mm, respectively. Moreover, both the Cardiovascular Health Study^2^ and the Health in Men Study^4^ showed that increased aortic diameter predicted incident intermittent claudication.

Firstly, the results of the present study confirm those of previous studies^4–6^ as we found that a smaller aortic diameter was associated with a higher prevalence of PAD in Chinese hypertensive adults. In the present study, LE-PAD was more prevalent in the lowest tertile when compared with the median and the adjusted OR was 1.58 (95% CI, 1.29–1.94). However, the present study differed from previous studies^2,4,6^ in that LE-PAD was found to be similarly prevalent in the highest tertile when compared with the median. Secondly, the adjusted OR was 0.92 (95% CI, 0.73–1.16), suggesting that a large aortic diameter is not associated with LE-PAD. This variation in results compared to previous studies^2,4,6^ could be attributed to the influence of racial differences.

The investigation of infrarenal aortic diameter has traditionally been pursued only in the context of abdominal aortic aneurysms (AAAs)^1,17^. While different suggestions for defining AAA do exist^8,18–23^, the definition of AAA as an infrarenal aortic diameter ≥3 cm is commonly accepted^1^. Furthermore, several studies^2–4^ have shown that the infrarenal aorta plays an important role in the prediction of cardiovascular and mortality risk in patients with AAA as well as those with small or enlarged non-aneurysmal aortas. Surprisingly, there is as yet no consensus or guidelines referring to the normal values of infrarenal aortic diameters in a Western population^1,17,18,24^. Similarly, this also applies to Chinese and Asian populations^25,26^, suggesting that more studies are needed in order to accurately determine the normal values. In an effort to avoid any problems related to the definition of normal values for infrarenal aortic diameters, the participants’ aortic diameters were classified into tertiles according to sex, as has been done in a previous study^6^. However, we defined LE-PAD as ABI ≤ 0.9 instead of using claudication, as this is more sensitive for the diagnosis of LE-PAD^16^ and as such differed from the methodologies employed by previous studies^2,4–6^. The sensitive analysis did not alter our findings, thus validating the stability and reliability of our results.

Considering that the present study involved a well-characterized, community-based design with a large population sample of 17279 hypertensive Chinese adults; the outcomes presented here are reliable and provide credible evidence of the association between abdominal aortic diameter and LE-PAD. Future prospective studies should aim to definitively evaluate the association between abdominal aortic diameter and the incidence and development of LE-PAD. Interpretation and application of our results should however take into consideration this study’s limitations. Firstly, this is a cross-sectional study of a defined hypertensive population and is not representative of the general population. Therefore, the association between abdominal aortic diameter and LE-PAD may not be casual. A prospective study in the general population is required to confirm the observed association. Secondly, the low prevalence of LE-PAD must be taken into account, as this may be indicative of differing population-specific characteristics and could lead to bias. Thirdly, the definition of LE-PAD is based only on ABI and not on claudication, which might lead to the absence of an association between the highest tertile of aortic diameter with LE-PAD. Therefore, further study is necessary to confirm the association.

In conclusion, small aortic diameter (as opposed to large aortic diameter) is significantly associated with LE-PAD in Chinese hypertensive adults. This association is consistent in both sexes as well as subgroups. Future, prospective studies are needed to confirm these findings and further explore the underlying mechanism in different populations.

## Electronic supplementary material

Table S1 and S2

## References

[1] Norman PE, Muller J, Golledge J (2011). The cardiovascular and prognostic significance of the infrarenal aortic diameter. J Vasc Surg.

[2] Freiberg MS (2008). Abdominal aortic aneurysms, increasing infrarenal aortic diameter, and risk of total mortality and incident cardiovascular disease events: 10-year follow-up data from the Cardiovascular Health Study. Circulation.

[3] Norman P, Le M, Pearce C, Jamrozik K (2004). Infrarenal aortic diameter predicts all-cause mortality. Arterioscler Thromb Vasc Biol.

[4] Lakshmanan R, Hyde Z, Jamrozik K, Hankey GJ, Norman PE (2010). Population-based observational study of claudication in older men: the Health in Men Study. Med J Aust.

[5] Rajkumar C, Bonapace S, Starr J, Radia M, Bulpitt CJ (1997). Association between abdominal aortic diameter and peripheral vascular disease. J Hum Hypertens.

[6] van den Bosch MA, van der Graaf Y, Eikelboom BC, Algra A, Mali WP (2001). Distal aortic diameter and peripheral arterial occlusive disease. J Vasc Surg.

[7] Huo Y (2015). Efficacy of folic acid therapy in primary prevention of stroke among adults with hypertension in China: the CSPPT randomized clinical trial. JAMA.

[8] Johnston KW (1991). Suggested standards for reporting on arterial aneurysms. Subcommittee on Reporting Standards for Arterial Aneurysms, Ad Hoc Committee on Reporting Standards, Society for Vascular Surgery and North American Chapter, International Society for Cardiovascular Surgery. J Vasc Surg.

[9] Bland JM, Altman DG (1986). Statistical methods for assessing agreement between two methods of clinical measurement. Lancet.

[10] Wei R (2015). Association of Resting Heart Rate with Infrarenal Aortic Diameter: A Cross Sectional Study in Chinese Hypertensive Adults. Eur J Vasc Endovasc Surg.

[11] He M (2012). Prevalence of unrecognized lower extremity peripheral arterial disease and the associated factors in chinese hypertensive adults. Am J Cardiol.

[12] Zheng M (2014). Age, arterial stiffness, and components of blood pressure in Chinese adults. Medicine (Baltimore).

[13] Song Y (2016). Independent and Joint Effect of Brachial-Ankle Pulse Wave Velocity and Blood Pressure Control on Incident Stroke in Hypertensive Adults. Hypertension.

[14] Yamashina A (2002). Validity, reproducibility, and clinical significance of noninvasive brachial-ankle pulse wave velocity measurement. Hypertens Res.

[15] Pan CR, Staessen JA, Li Y, Wang JG (2007). Comparison of three measures of the ankle-brachial blood pressure index in a general population. Hypertens Res.

[16] Rooke TW (2011). 2011 ACCF/AHA Focused Update of the Guideline for the Management of Patients With Peripheral Artery Disease (updating the 2005 guideline): a report of the American College of Cardiology Foundation/American Heart Association Task Force on Practice Guidelines. J Am Coll Cardiol.

[17] Sconfienza LM (2013). When the diameter of the abdominal aorta should be considered as abnormal? A new ultrasonographic index using the wrist circumference as a body build reference. Eur J Radiol.

[18] W. A (2008). How to define an abdominal aortic aneurysm – influence on epidemiology and clinical practice. Scand J Surg.

[19] McGregor JC, Pollock JG, Anton HC (1975). The value of ultrasonography in the diagnosis of abdominal aortic aneurysm. Scott Med J.

[20] Sterpetti AV (1987). Factors influencing enlargement rate of small abdominal aortic aneurysms. J Surg Res.

[21] Collin JAL, Walton J, Lindsell D (1988). Oxford screening programme for abdominal aortic aneurysm in men aged 65 to 74 years. Lancet.

[22] Sconfienza LM (2013). When the diameter of the abdominal aorta should be considered as abnormal? A new ultrasonographic index using the wrist circumference as a body build reference. European Journal of Radiology.

[23] Wanhainen A, Themudo R, Ahlstrom H, Lind L, Johansson L (2008). Thoracic and abdominal aortic dimension in 70-year-old men and women–a population-based whole-body magnetic resonance imaging (MRI) study. J Vasc Surg.

[24] Rogers IS (2013). Distribution, determinants, and normal reference values of thoracic and abdominal aortic diameters by computed tomography (from the Framingham Heart Study). Am J Cardiol.

[25] Joh JH, Ahn HJ, Park HC (2013). Reference diameters of the abdominal aorta and iliac arteries in the Korean population. Yonsei Med J.

[26] Poon JTC, Cheng SWK, Wong JSW, Ting ACW (2010). Prevalence of abdominal aortic aneurysm in Chinese patients with severe coronary artery disease. Anz Journal of Surgery.

