# Supplementary material for: Small infrarenal aortic diameter associated with lower-extremity peripheral artery disease in Chinese hypertensive adults
Source: Sci Rep. 2017 Nov 6;7:14547. doi: 10.1038/s41598-017-12587-x (PMC5674057; doi:10.1038/s41598-017-12587-x)
Supplement: Supplementary file 1 — Table S1 and S2 [file 41598_2017_12587_MOESM1_ESM.doc]

**Full title: Small infrarenal aortic diameter associated with lower-extremity peripheral artery disease in Chinese hypertensive adults**

Jie Liu^1,2,+^; Xin Jia^1,+^; Senhao Jia ^1,+^; Xianhui Qin^3^; Tao Zhang^4^; Lishun Liu^5^; Haibo Li^5^, Dan Rong^1^; Ziyi Zhou^5^; Yuxiang Song^1^; Shangwei Zuo^1^; Chen Duan^1^; Zhongyin Wu^1^; Ren Wei^1^; Yangyang Ge^1^; Xian Wang^6,7^; Wei Kong^6,7^; Xiping Xu^3^; Raouf A. Khalil^2^; Yong Huo^8,*^ &Wei Guo^1,*^

**Running Title: Association of aortic diameter with peripheral artery disease**

^1^ Department of Vascular and Endovascular Surgery, Chinese PLA General Hospital, Beijing, China

^2^Vascular Surgery Research Laboratories, Division of Vascular and Endovascular Surgery, Brigham and Women's Hospital, Harvard Medical School, Boston, Massachusetts, United States

^3^ National Center for Clinical Research in Kidney Disease, Guangdong Institute of Nephrology, Southern Medical University, Guangzhou, China

^4^ Department of Vascular Surgery, Peking University People’s Hospital, Beijing, China

^5^ Institute of Biomedicine, Anhui Medical University, Hefei, China

^6^ Department of Physiology and Pathophysiology, School of Basic Medical Sciences, Peking University, Beijing, China

^7^Key Laboratory of Molecular Cardiovascular Science, Ministry of Education, Beijing, China

^8^ Department of Cardiology, Peking University First Hospital, Beijing, China

*Corresponding author

+These authors contributed equally to this work

Correspondence and reprint requests should be addressed to:

Wei Guo, MD

Department of Vascular and Endovascular Surgery, Chinese PLA General Hospital, 28 Fuxing Rd, Haidian District, Beijing 100853, China

Email: [pla301dml@vip.sina.com](mailto:pla301dml@vip.sina.com)

Phone: 86-10-66938049

Yong Huo, MD

Department of Cardiology, Peking University First Hospital, No. 8 Xishiku St, XichengDistrict, Beijing 100034, China

Email:huoyong@263.net.cn

Conflict of interest: None

**Table S1. OR (95% CI) for lower-extremity peripheral artery disease in relation to tertiles of the aortic diameter**

| Overall | Crude |  |  | Model I |  |  | Model II |  |
| --- | --- | --- | --- | --- | --- | --- | --- | --- |
|  | OR, 95% CI | P-value |  | OR, 95% CI | P-value |  | OR, 95% CI | P-value |
| First vs. second tertile | 1.89 (1.55, 2.29) | <0.001 |  | 1.86 (1.53, 2.26) | <0.001 |  | 1.58 (1.29, 1.94) | <0.001 |
| Third vs. second tertile | 0.91 (0.72, 1.13) | 0.38 |  | 0.87 (0.69, 1.09) | 0.22 |  | 0.92 (0.73, 1.16) | 0.49 |

OR, Odds ratio. CI, confidence interval.

Distal Aortic Diameter, median (range), mm. men: lowest tertile, 11.90 ( 4.70-12.90) mm; middle tertile, 13.80 (13.00-14.55) mm; 15.60 (14.60-51.30); highest tertile, 15.60 (14.60-51.30) mm. women: lowest tertile, 10.60 ( 5.20-11.50) mm; middle tertile, 12.30 (11.60-13.15) mm; highest tertile, 14.10 (13.20-30.50) mm.

Crude, not adjusted; Model I adjusted for sex, age and body mass index; Model II adjusted for sex, age, body mass index, heart rate, systolic blood pressure, diastolic blood pressure, cigarette smoking, folic acid supplementation, level of total cholesterol, triglycerides, HDL-C, fasting glucose, creatinine, uric acid, homocysteine, and MTHFR C677T polymorphisms.

**Table S2.** Multiple logistic regression analysis for LE-PAD in relation to tertiles of the aortic diameter according to different subgroups

| Subgroups | Number of participants | First vs.  second tertile |  |  | Third vs.  second tertile |  |  | P-value for interaction |
| --- | --- | --- | --- | --- | --- | --- | --- | --- |
|  | n (%) | OR, 95% CI | P-value |  | OR, 95% CI | P-value |  |  |
| Sex |  |  |  |  |  |  |  | 0.763 |
| Male | 6590 (38.1%) | 1.67 (1.19, 2.34) | 0.003 |  | 0.96 (0.65, 1.41) | 0.82 |  |  |
| Female | 10689 (61.9%) | 1.55 (1.21, 1.99) | <0.001 |  | 0.89 (0.67, 1.18) | 0.40 |  |  |
| Age |  |  |  |  |  |  |  | 0.157 |
| <65 | 8834 (51.2%) | 1.22 (0.87, 1.73) | 0.25 |  | 0.96 (0.67, 1.38) | 0.84 |  |  |
| ≥65 | 8445 (48.8%) | 1.77 (1.38, 2.27) | <0.001 |  | 0.89 (0.66, 1.20) | 0.45 |  |  |
| BMI, kg/m^2^ |  |  |  |  |  |  |  | 0.067 |
| <25 | 9251 (53.5%) | 1.83 (1.36, 2.46) | <0.001 |  | 0.97 (0.68, 1.38) | 0.85 |  |  |
| ≥25 | 8028 (46.5%) | 1.33 (1.01, 1.77) | 0.04 |  | 0.86 (0.64, 1.16) | 0.33 |  |  |
| MTHFR C677T polymorphisms |  |  |  |  |  |  |  | 0.790 |
| CC | 4834 (28.0%) | 1.92 (1.28, 2.86) | 0.002 |  | 1.10 (0.70, 1.73) | 0.69 |  |  |
| CT | 8427 (48.8%) | 1.55 (1.17, 2.04) | 0.002 |  | 0.88 (0.64, 1.21) | 0.44 |  |  |
| TT | 4018 (23.2%) | 1.32 (0.86, 2.03) | 0.20 |  | 0.82 (0.51, 1.31) | 0.41 |  |  |
| Smoking |  |  |  |  |  |  |  | 0.522 |
| Never | 11861 (68.7%) | 1.55 (1.21, 2.00) | <0.001 |  | 0.90 (0.68, 1.19) | 0.46 |  |  |
| Former | 1877 (10.9%) | 2.28 (1.17, 4.47) | 0.02 |  | 0.99 (0.43, 2.25) | 0.98 |  |  |
| Current | 3541 (20.5%) | 1.56 (1.04, 2.34) | 0.03 |  | 0.97 (0.62, 1.54) | 0.91 |  |  |
| Folic acid supplementation |  |  |  |  |  |  |  | 0.452 |
| No | 10488 (60.70%) | 1.55 (1.21, 2.00) | <0.001 |  | 0.83 (0.62, 1.11) | 0.21 |  |  |
| Yes | 6791 (39.30%) | 1.65 (1.18, 2.31) | 0.003 |  | 1.09 (0.76, 1.58) | 0.62 |  |  |

LE-PAD, lower-extremity peripheral artery disease; BMI, Body mass index; OR, odds ratios. CI, confidence interval.

Distal Aortic Diameter, median (range), mm. men: lowest tertile, 11.90 ( 4.70-12.90) mm; middle tertile, 13.80 (13.00-14.55) mm; 15.60 (14.60-51.30); highest tertile, 15.60 (14.60-51.30) mm. women: lowest tertile, 10.60 ( 5.20-11.50) mm; middle tertile, 12.30 (11.60-13.15) mm; highest tertile, 14.10 (13.20-30.50) mm.

The regression analysis was adjusted for sex, age, body mass index, heart rate, systolic blood pressure, diastolic blood pressure, cigarette smoking, folic acid supplementation, level of total cholesterol, triglycerides, HDL-C, fasting glucose, creatinine, uric acid, homocysteine, and MTHFR C677T polymorphisms.
